# Soluble epoxide hydrolase limits mechanical hyperalgesia during inflammation

**DOI:** 10.1186/1744-8069-7-78

**Published:** 2011-10-04

**Authors:** Christian Brenneis, Marco Sisignano, Ovidiu Coste, Kai Altenrath, Michael J Fischer, Carlo Angioni, Ingrid Fleming, Ralf P Brandes, Peter W Reeh, Clifford J Woolf, Gerd Geisslinger, Klaus Scholich

**Affiliations:** 1 Pharmazentrum Frankfurt/ZAFES, Institute of Clinical Pharmacology, Johann Wolfgang Goethe-University, Frankfurt, Germany; 2 F. M. Kirby Neurobiology Center, Department of Neurology, Children's Hospital Boston, Boston, MA, USA; 3 Department of Pharmacology, University of Cambridge, Cambridge, UK; 4 Institute for Vascular Signalling, ZAFES, Faculty of Medicine, Johann Wolfgang Goethe-University, Frankfurt, Germany; 5 Institute for Cardiovascular Physiology, ZAFES, Faculty of Medicine, Johann Wolfgang Goethe-University, Frankfurt, Germany; 6 Department of Physiology and Pathophysiology, Friedrich-Alexander-University Erlangen-Nürnberg, Erlangen, Germany

**Keywords:** sEH, EET, CYP450, nociceptors, TRPA1, hyperalgesia

## Abstract

**Background:**

Cytochrome-P450 (CYP450) epoxygenases metabolise arachidonic acid (AA) into four different biologically active epoxyeicosatrienoic acid (EET) regioisomers. Three of the EETs (i.e., 8,9-, 11,12- and 14,15-EET) are rapidly hydrolysed by the enzyme soluble epoxide hydrolase (sEH). Here, we investigated the role of sEH in nociceptive processing during peripheral inflammation.

**Results:**

In dorsal root ganglia (DRG), we found that sEH is expressed in medium and large diameter neurofilament 200-positive neurons. Isolated DRG-neurons from sEH^-/- ^mice showed higher EET and lower DHET levels. Upon AA stimulation, the largest changes in EET levels occurred in culture media, indicating both that cell associated EET concentrations quickly reach saturation and EET-hydrolyzing activity mostly effects extracellular EET signaling. *In vivo*, DRGs from sEH-deficient mice exhibited elevated 8,9-, 11,12- and 14,15-EET-levels. Interestingly, EET levels did not increase at the site of zymosan-induced inflammation. Cellular imaging experiments revealed direct calcium flux responses to 8,9-EET in a subpopulation of nociceptors. In addition, 8,9-EET sensitized AITC-induced calcium increases in DRG neurons and AITC-induced calcitonin gene related peptide (CGRP) release from sciatic nerve axons, indicating that 8,9-EET sensitizes TRPA1-expressing neurons, which are known to contribute to mechanical hyperalgesia. Supporting this, sEH^-/- ^mice showed increased nociceptive responses to mechanical stimulation during zymosan-induced inflammation and 8,9-EET injection reduced mechanical thresholds in naive mice.

**Conclusion:**

Our results show that the sEH can regulate mechanical hyperalgesia during inflammation by inactivating 8,9-EET, which sensitizes TRPA1-expressing nociceptors. Therefore we suggest that influencing the CYP450 pathway, which is actually highly considered to treat cardiovascular diseases, may cause pain side effects.

## Background

Inflammatory responses after tissue damage or infection cause the release of arachidonic acid (AA) and its subsequent metabolism to biologically active lipids in activated immune cells and hyperactive neurons [1]. Free AA is a major substrate for cyclooxygenases (COX), lipoxygenases (LOX) and cytochrome P450 (CYP450) epoxygenases which metabolize it to prostanoids, leukotrienes and epoxyeicosatrienoic acids (EETs) and hydroxyeicosatetranoic acids (HETES), respectively [2-4]. At the site of injury and in the CNS prostanoids and leukotrienes are inflammatory and pain mediators that attract and activate immune cells as well as directly sensitize nociceptive neurons [5,6].

Recently, those EETs involved in vascular homeostasis and coronary physiology have been shown also to influence nociceptive processing [7,8]. EETs either bind intracellular targets, are released to act as auto- or paracrine mediators or are stored in cell membranes esterified to phospholipids [9]. They activate PPARγ or the cAMP/PKA pathway and modulate and/or activate a variety of channels including several transient receptor potential (TRP) channel isoforms, large-conductance Ca^2+^-activated K^+ ^channels (BK_(Ca)_) and L-type voltage gated calcium channels (Ca_(v)_) [9].

Three EETs (8,9-, 11,12- and 14,15-EET) are metabolized by soluble epoxide hydrolase (sEH) to their corresponding dehydro metabolites (DHET), which are thought to be less active [10]. Increasing EET bioavailability by blocking sEH activity or expression can be used to study the actions of EETs *in vivo*. sEH inhibition strongly reduces nociceptive responses, suppresses COX-2 expression and up-regulates the acute neurosteroid-producing gene StARD1, when applied either topically to the inflammation site or injected intrathecally in a model of inflammatory and neuropathic pain [11,12]. Furthermore, a conditional knockout of cytochrome P450 reductase (CRP) in CNS neurons, which blocks CYP450 activity, largely abolishes morphine-induced anti-nociception, suggesting that EETs have an anti-nociceptive activity downstream of the μ-opioid receptor [7].

EETs can however, directly activate TRPV4 channels as well as elicit rapid membrane insertion of TRPV4 and TRPC6 channels [13-15] which may be pro-nociceptive. In DRG-neurons, calcium influx after TRP-channel opening leads to activation of signaling molecules like p38 mitogen-activated protein-kinase, which sensitize the neurons for further excitation [16]. TRPV4 and TRPC6, as other TRP- family channels including, TRPV1 and TRPA1, mediate thermal and mechanical hyperalgesia in various disease models [17,18]. However, the mechanism how they are activated by specific endogenous agonists is largely unknown.

We have now investigated EETs in nociceptive processing by phenotyping mice with targeted deletion of sEH, and find that the EETs metabolized by sEH sensitize TRPA1 expressing DRG neurons and that sEH^-/- ^mice show an increased mechanical hyperalgesia.

## Results

### sEH is expressed in primary afferent neurons

**Figure 1:**
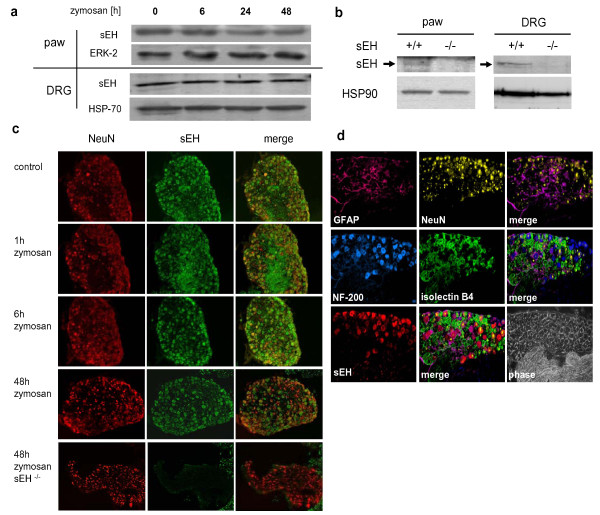
**Expression pattern of sEH in primary afferent neurons**. (A) Representative western blot analysis of sEH-expression during zymosan induced inflammation in paw and DRG-tissue 0 - 48h after zymosan injection. (B) Western blot detection of sEH expression in tissues from dorsal root ganglia and paw oedema tissue of wild type and sEH-deficient mice 48h after zymosan injection. Arrowhead indicates a signal appearing only in wild- type tissue at approximately 58 kDa. (C) Expression pattern of sEH in dorsal root ganglia neurons. Immunofluorescence staining of L4 or L5 dorsal root ganglia cryosections during zymosan induced inflammation (0h, 1h, 6h and 48h zymosan) was performed with antibodies against sEH (green) and NeuN (red). At time point 48h post zymosan injection wildtype and sEH-deficient DRG-tissue were compared. (D) Selective sEH expression in NF200 positive neurons. Combination of conventional immunofluorescence staining for sEH as in (C) with the MELC technology using 7AAD and FITC-labeled primary antibodies against NeuN, glial fibrillary acidic protein (GFAP), isolectin B4 (IB4) and neurofilament 200 (NF200).

To determine if and how sEH could potentially contribute to nociceptive processing we first analysed the cellular distribution of sEH by Western blotting and immunohistochemistry in edematous paw and DRG tissue up to 48 h after an intraplantar zymosan injection. Western blotting of tissue from wild- type mice revealed a 58 kDa band which corresponds to the predicted size of sEH but was not regulated in paw or DRG tissue (Figure 1A). The band was specific for sEH, since it was absent in the sEH^-/- ^animals (Figure 1B). Comparison of tissue sections from wild- type and sEH^-/- ^animals by immunohistochemistry showed specific sEH staining in the DRG (Figure 1C). Notably, the distribution and localization of sEH immunoreactivity was not altered in DRGs after zymosan treatment (Figure 1C). As indicated by co-staining with NeuN, sEH expression in the DRG was restricted to a subset of neurons. To determine which cell types express sEH in DRGs, we combined conventional immunohistochemistry with the Multi-epitope ligand cartography-(MELC)-system which allows repeated immunostaining using FITC-labelled primary antibodies against several cell type-specific markers [19,20]. We compared sEH expression with staining for NeuN as a marker for all neurons, GFAP as a marker for satellite glial cells, IB4 as a marker for unmyelinated small diameter nociceptive neurons and NF200 as a marker for myelinated medium and large diameter neurons. The co-localisation pattern indicated that sEH is predominantly expressed in NF200-positive neurons (Figure 1D). These medium and large sized neurons have myelinated axons forming Aβ-and Aδ-fibers, with the latter predominatly involved in fast nociception [21]. Further, a communication between innocuous Aβ- and noxious C- or Aδ-fibers can play a role in the development of allodynia [22].

### sEH activity in isolated DRG neurons and its contribution to EET levels during inflammation

**Figure 2:**
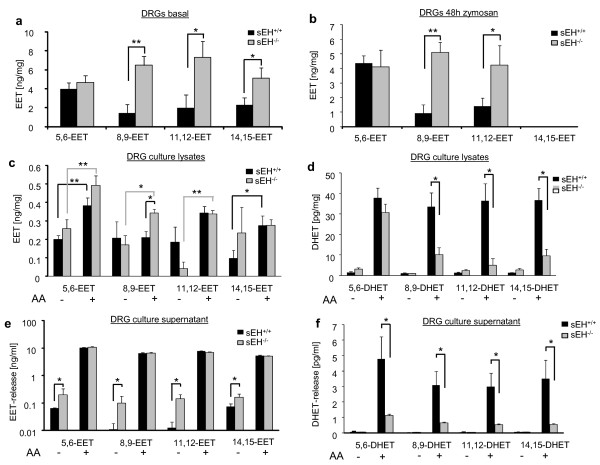
**Contribution of sEH to EET release from sensory neurons and DRG tissue levels during inflammation**. (A) Basal EET levels extracted from tissues of DRGs from L4 and L5 segments from wild type and sEH deficient mice were determined by LC-MS/MS analysis. (B) EET-tissue levels in DRGs 48h after intraplantar zymosan injection. Data shown represent the mean ± SEM from 6 animals. (C) EET and DHET (D) levels in lysates of sensory neurons. Neuron enriched cultures from DRGs of wild- type and sEH-deficient mice were stimulated with 0.5 μM arachidonic acid and incubated for 2 h until extraction of EETs from cells. Data shown represent the mean ± SEM from 5 culture dishes. (E) EET and DHET (F) release from sensory neurons.. EETs were extracted from culture media of cells used in panel (C). Data shown represent the mean ± SEM from 5 culture dishes. Student's *t *test: *, *p *≤ 0.05; **, *p *≤ 0.01.

To investigate if sEH contributes to steady state EET levels *in vivo*, we extracted lipids form DRG tissue of naive wild type and sEH^-/- ^mice. EET quantification by LC-MS/MS analysis revealed that deletion of sEH increased 8,9-EET, 11,12-EET and 14,15-EET tissue concentrations (Figure 2A) while 5,6-EET levels were unaffected. DHETs were not detected in DRG tissue. To test if sEH expression is regulated during peripheral inflammation and if differences in EET levels become more pronounced, we analysed DRG tissue samples 48h after intraplantar zymosan injection. We found no difference in 5,6-EET, 8,9-EET and 11,12-EET tissue concentrations (Figure 2B). However, 14,15-EET concentrations were reduced below the detection limit. We next investigated the functional contribution of sEH to EET hydrolysis in DRG neurons by comparing basal EET synthesis and that after stimulation with 0.5 μM AA in DRGs from wild type and sEH deficient mice. In neuronal cell lysates, we detected only a small increase in the levels of some EETs, and only 8,9-EET was elevated in sEH^-/- ^compared to sEH^+/+ ^cells (Figure 2C). However, the level of DHET markedly increased when AA was added, and this was significantly higher in wild type than in sEH^-/- ^cells (Figure 2D).

In contrast, we found dramatically elevated EET levels (around 100-fold) after AA stimulation in the cell culture medium (Figure 2E). This indicates that cell associated EET concentrations quickly reach saturation and that higher AA availability increases EET release more than its levels within neurons. Interestingly, the extracellular DHET concentrations were strongly induced upon AA stimulation, an effect which was markedly reduced in sEH^-/- ^neurons (Figure 2F). These data suggest that after increases in AA availability, EETs are released from sensory neurons and that a deficiency in cellular hydrolysis by deletion of sEH amplifies this effect.

### Role of sEH on EET and prostaglandin levels at the inflammation side

**Figure 3:**
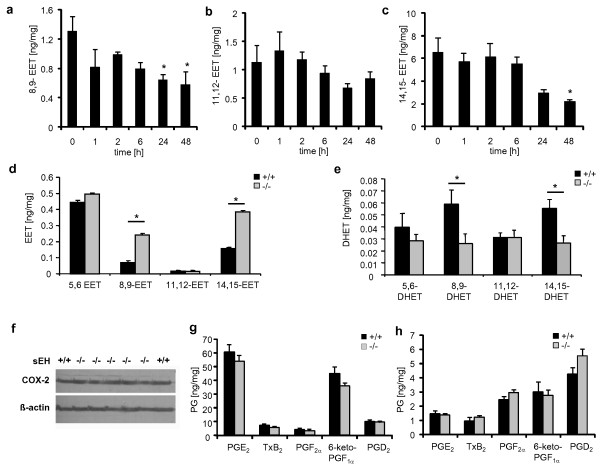
**Consequences of sEH deletion on EET and prostaglandin levels in the inflamed paw**. (A-C) Changes in EET levels during zymosan induced inflammation. Levels of 8,9- (A), 11,12-(B) and 14,15-EET (C) levels were determined by LC-MS/MS in paw tissues at different time points after zymosan injection. (D) Changes in EET and DHET (E) levels after sEH deletion. Four different regioisomers were determined by LC-MS/MS analysis in paw tissue from wild type and sEH^-/- ^mice 48 h after intraplantar zymosan injection. Data shown represent the mean ± SEM from tissues of 4-5 animals. Student's *t *test: *, *p *≤ 0.05. (F) Effect of sEH deletion on COX-2 expression. COX-2 protein levels were determined by western blot in paw tissues from wild- type and sEH deficient mice 48 h after intraplantar zymosan injection. (G) Effect of sEH deletion on prostaglandin synthesis in the inflamed paw. Prostaglandins were determined 48h after zymosan injection by LC-MS/MS analysis. Data shown represent the mean ± SEM from tissues of 3-5 animals. (H) Effect of sEH deletion on prostaglandin synthesis in the spinal cord. Prostaglandins were determined from lumbal spinal cord tissue 48h after zymosan injection. Data shown represent the mean ± SEM from tissues of 4 animals.

To elucidate if EET levels differ at the site of inflammation, we determined their levels in zymosan-inflamed paws. First, we compared EET levels in wild- type mice at the onset (1-2 h), peak (6 h) and recovery phases (24-48 h) of inflammation after zymosan injection. We found, no significant changes in EET levels up to 6 hours after zymosan injection (Figure 3A-C). However, at later time points i.e., during the recovery phase, 8,9-EET and 14,15-EET levels decreased significantly (Figure 3A, C). Next, we determined how EET levels were altered by sEH deletion, 48 h after zymosan injection. In sEH^-/- ^mice, 8,9- and 14,15-EET levels were significantly increased while the corresponding DHETs were significantly reduced (Figure 3D, E). 11,12-EET levels and 5,6-EET were unaffected.

Recent studies suggest that sEH inhibition has an antinociceptive effect in LPS-induced hyperalgesia and in streptozocin-induced diabetic neuropathy [11,12,21,22]. One explanation for these findings is a direct anti-inflammatory effect of the sEH inhibitors followed by suppression of COX-2 expression in the inflamed paw and spinal cord [10,11]. Since COX-2 is also strongly upregulated in the zymosan model for up to 96 hours [12] and hyperalgesia in this model strongly depends on its activity [23,24], we examined COX-2 expression and prostaglandin synthesis in the sEH^-/- ^mice. However, 48 h after zymosan injection, a time point at which COX-2 expression remains upregulated in inflamed paws, no differences in COX-2 expression or PGE_2_, PGD_2_, TXB_2_, PGF_2α _or 6-keto-PGF_1α _levels at the site of inflammation or in the spinal cord were observed in wild type and sEH knockout mice (Figure 3F-H).

### Effects of EETs on nociceptive neurons

**Figure 4:**
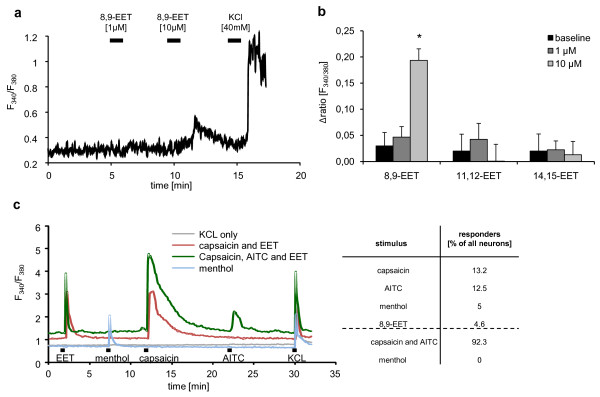
**Activation of primary afferent neurons by 8,9-EET**. (A) 8,9-EET induces direct calcium influx in sensory neurons. Calcium concentrations were monitored by ratiometric imaging using fura-2 in cultivated neurons from DRGs of adult wild- type mice. Shown is a representative trace. 40 mM KCL solution was used to identify all viable neurons (B). Average values of peak normalized to baseline ratios of experiments shown in panel B. Data shown represent the mean ± SEM from 12 experiments. Student's *t *test: *, *p *≤ 0.05. (C) Phenotypic characterisation of 8,9-EET responsive neurons. Cultivated DRG neurons were stimulated with 10 μM 8,9-EET, 200 μM menthol, 300 nM capsaicin, 100 μM AITC and 40 mM KCL for 10 sec. Representative traces are shown. Relative amount of cells grouped according to their responsiveness to 8,9-EET and TRP-agonists. Percent values were calculated from 280 KCl responsive neurons. Values below the dotted line are the percentage of all 8,9-EET responding cells.

Next we determined whether increases in EET levels in sensory ganglia after sEH deletion alters nociceptive neurons. To do this, we applied 8,9-, 11,12- or 14-15-EET to cultured DRG neurons and determined calcium fluxes using fura-2-AM. At 1 μM none of the EETs induced significant changes in intracellular calcium levels. However, 10 μM 8,9-EET, but not 11,12- or 14-15-EET, increased intracellular calcium concentrations when applied for 10 sec (Figure 4A, B). This calcium transient was observed in 4.7% of all neurons as determined by the responsiveness of the cells to 40 mM KCl. We further characterized the responding cells by co-stimulation with menthol, capsaicin and AITC to identify TRPM8, TRPV1 and TRPA1 expressing neurons respectively (Figure 4C). 8,9-EET responding neurons were predominantly capsaicin- and AITC-sensitive (92.3%) showing that these cells are nociceptive neurons.

**Figure 5:**
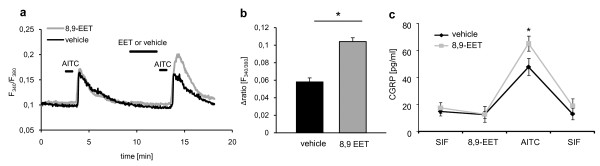
**8,9-EET sensitizes AITC induced TRPA1 response**. (A) Representative calcium imaging experiment of 8,9-EET dependent TRPA1-sensitization. DRG cells were stimulated with AITC twice (50 μM, 15 sec) with 10 minutes interval. 1 μM 8,9-EET or vehicle was perfused for two minutes prior to the second AITC stimulation. (B) Statistical analysis comparing the amplitudes of AITC induced TRPA1 response. Data shown represent the mean ± SEM of 21-23 cells. Student's *t *test: *, *p *≤ 0.05 (C) 8,9-EET sensitizes AITC induced CGRP release from sciatic nerve axons. Freshly isolated sciatic nerves were incubated with 10 μM 8,9-EET (ipsilateral) or 3.2% EtOH (vehicle, contralateral) and stimulated with 50 μM AITC. CGRP was determined by ELISA from extracellular solutions. Data shown represent the mean ± SEM from nerves of 3-4 animals. Two way ANOVA with Bonferroni post test: *, *p *≤ 0.05.

Next, we determined whether or not low concentrations of 8,9-EET contribute to the sensitization of nociceptive neurons. Therefore, we studied the effect of 1 μM 8,9-EET on AITC-induced calcium increases. We found that 8,9-EET significantly increased the amplitude of AITC evoked calcium increases in cultured DRG neurons (Figure 5A, B). To further investigate nociceptor activation by 8,9-EET, we measured the release of neuropeptide from freshly isolated sciatic nerves [25]. TRPA1 channels are expressed along peptidergic nerve fibers and neuropeptide release can be stimulated by incubation with AITC [26]. To test, whether 8,9-EET can sensitize neuropeptide release from those fibers, we incubated sciatic nerves with 10 μM 8,9-EET or with 3.2% ethanol (vehicle) before stimulation with 50 μM AITC. While 8,9-EET had no direct effect, it significantly sensitized AITC-induced CGRP release from the nerves (Figure 5C).

### Effects of EETs and sEH deletion on nociceptive thresholds during inflammation

**Figure 6:**
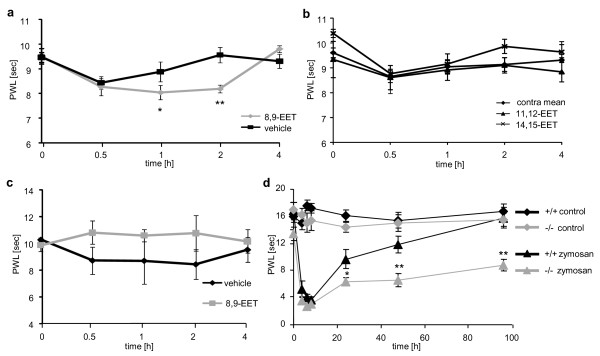
**Effects of EETs and sEH deletion on mechanical pain thresholds**. (A) 8,9-EET induces mechanical hyperalgesia. 20 μl of 10 μM 8,9-EET was injected intraplantar and mechanical thresholds were determined by the Dynamic Plantar Test. Control animals received vehicle solution containing the corresponding volumes of ethanol (3.2% v/v) and were tested in parallel. Data shown represent the mean ± SEM from 8 animals per group. Two way Anova with Bonferroni post test: *, *p *≤ 0.05; **, *p *≤ 0.01. (B) Same experiments than in (A) but other EET regioisomers were used. (C) Thermal thresholds after 8,9-EET injection. 20 μl of 10 μM 8,9-EET or vehicle was injected and thermal thresholds were determined by the Hargreaves test. Data shown represent the mean ± SEM from 4 animals. (D) Effect of sEH deletion on mechanical hyperalgesia after zymosan injection. Mechanical thresholds of both hind paws were tested by the dynamic plantar test after unilateral intraplantar zymosan injection and compared between sEH^-/- ^mice and wild type C57BL/6 controls. Data shown represent the mean ± SEM from 8-9 animals per group. Two way ANOVA with Bonferroni post test: **, *p *≤ 0.01.

TRPA1-expressing neurons appear to have an important contribution to the development of mechanical hyperalgesia [27-29]. To test whether 8,9-EET, which activates and sensitizes TRPA1-expressing neurons, also induces mechanical hyperalgesia, we injected 10 μM of 8,9-EETs intraplantarily and determined mechanical thresholds using the Dynamic Plantar Aesthesiometer. Injection of vehicle (3.2% ethanol (v/v)) reduced mechanical thresholds 30 minutes after injection (Figure 6A). However, this effect recovered after 1 hour while mice receiving 8,9-EET showed significantly lower mechanical thresholds for up to 2 hours. In contrast, injections of 11,12- and 14,15-EET had no effect on nociceptive behavior (Figure 6B).

Interestingly, after 8,9-EET injection we did not detect any significant differences in thermal thresholds, indicating that EETs affect noxious mechanical perceptions specifically (Figure 6C). To investigate the role of sEH in nociceptive processing, we used the mice with a targeted gene deletion of sEH, which exhibit much higher 8,9-EET levels in DRGs after intraplantar injection of zymsoan A. Within the first 10 hours, wild type and sEH^-/- ^mice developed a comparable degree of hyperalgesia (Figure 6D). However, from 24 to 96 hours after the zymosan-injection, sEH-deficient mice exhibited strongly reduced mechanical thresholds compared to the WT mice, indicating that deletion of sEH prolongs mechanical hyperalgesia during peripheral inflammation.

Taken together, these data suggests that 8,9-EET sensitizes TRPA1 expressing primary afferent neurons and that this may reduce mechanical thresholds. Moreover, sEH^-/- ^animals which have significantly elevated 8,9-EET-levels in DRGs and paw show elevated mechanical hyperalgesia during zymosan induced inflammation.

## Discussion

In the present study we used a genetic mouse model to characterise the role of sEH in nociceptive processing. Our findings suggest that the sEH is expressed in a subpopulation of myelinated primary sensory neurons. There, it negatively regulates steady state EET tissue levels as well as release of EETs from neurons. Specifically we show that 8,9-EET activates TRPA1-expressing neurons and sensitizes neuropetide release from TRPA1-expressing fibers. Finally, we find that 8,9-EET can induce mechanical hyperalgesia and that sEH deletion prolongs mechanical hyperalgesia during inflammation.

sEH exhibits lipid phosphatase as well as epoxide hydrolase activity, with the latter resulting in rapid inactivation of EETs and epoxyoctadecenoic acids (EpOMEs) linoleic acid metabolites [11,30]. Although a putative lipid phosphatase activity may have an impact on nociceptive processing, we focused on the epoxide hydrolase activity of sEH and on EETs/DHETs since they mediate most sEH functions in the cardiovascular system [9,31].

The EETs are generally attributed with anti-inflammatory effects, largely on the basis of the fact that 11,12-EET can inhibit the IκB kinase and NF-κB signaling [32]. However, although 11,12-EET appears to be the most potent with respect to anti-inflammatory, anti-migratory, and pro-fibrinolytic effects [33], it has also been reported to increase COX2 expression in endothelial cells, a phenomenon linked to angiogenesis [34]. This finding is of relevance, as one major consequence of immune cell activation after zymosan injection is induction of COX-2 expression at the inflammation site and in the spinal cord to sensitize nociceptive processing [23,24]. However, we found no obvious change in COX-2 expression or in prostaglandin synthesis in paw tissue from zymosan-treated sEH^-/- ^mice that could be attributed to the higher EET levels in these animals. Our results suggest that sEH deletion, which mainly increases levels of 8-9- and 14,15-EET in the tissue studied, does not alter zymosan-induced inflammatory hyperalgesia by an inhibition of prostaglandin synthesis. These findings contrast with those made by Incoeglu et al. who described that inhibition of sEH reduces PGD_2 _synthesis and hyperalgesia in a LPS model [11]. While these reports are difficult to reconcile, it is possible that differences in the mechanisms of immune cell activation between the two models may be a determinant factor since LPS and zymosan selectively activate toll like receptor 4 (TLR-4) and TLR-2, respectively [35] leading to expression and release of a different set of proinflammatory mediators, which may be differentially affected by EETs.

All studies reporting antinociceptive effects of sEH are based on a pharmacological inhibition of the enzyme [10,36,37]. The model used in the present investigation was that of *Ephx *gene deletion resulting in the loss of the N-terminal lipid phosphatase activity and C-terminal soluble epoxide hydrolase activity. Thus, one major difference between our study and those performed previously is the fact that the sEH-associated lipid phosphatase activity was also inhibited. As no endogenous substrates or pharmacological inhibitors have been identified that target sEH lipid phosphatase activity, it is currently not possible to distinguish between the contributions of the lipid phosphatase and the epoxide hydrolase activities to the phenotype of sEH^-/- ^mice [38]. Another possible explanation for the differences between this and previous studies could be related to off-target effects of the urea-based inhibitors used in the pharmacological studies. Although specificity for sEH over other epoxide hydrolases such as mEH is generally good for most inhibitors, some compounds including 12-(3-adamantan-1-yl-ureido)-dodecanoic acid (AUDA) can also activate PPAR□, which represses COX-2 expression [39,40].

We did not detect the sEH in invading immune cells within inflamed paw tissue. In contrast, we found prominent expression of sEH in primary afferent neurons in the DRG. It seems that also other components of the EET generating/metabolising pathway are present in sensory neurons. The epoxygenases CYP2J3 and CYP2J4 are expressed together with the sEH in trigeminal ganglia, indicating that the CYP450/sEH pathway is a common, integral component of peripheral sensory neuron signaling [41].

In the nociceptive models used in this study mechanical or heat stimuli are applied to the plantar surface of the hind paw. Here, nociception is processed by small and medium diameter DRG-neurons with C- and A-δ fibres. We found that the sEH was mainly expressed in NF200 positive large and medium diameter DRG neurons. Most of these cells transmit proprioceptive and low threshold mechanical but not noxious sensations. However, we found that sEH deficient neurons have a higher steady state release of EET into the extracellular medium. Further, we showed that an increase of AA bioavailability predominately affects EET release instead of intracellular accumulation. This implies that the sEH present in myelinated neurons may also affect C-fibre neurons by paracrine signaling. Neuronal activity as well as inflammation activates PLA_2 _in primary afferents causing increased synthesis of eicosanoids. The restricted expression pattern of sEH in a subset of DRG neurons and its hydrolyzing activity on EET, preventing their release, suggests that the sEH may act to limit EET signaling to nociceptors.

Using cellular imaging experiments we identified 8,9-EET as the sEH substrate most likely to sensitize nociceptor function. 8,9-EET induced a direct calcium influx in ≈5% of sensory neurons. Co-stimulation with different TRP-channel agonists revealed that 8,9-EET only activates a subset of capsaicin responsive nociceptors and not large non nociceptive neurons. Further, we found that within the capsaicin-responsive group most were also AITC-responsive (TRPA1-positive neurons) (92.3% positive cells). Notably, Kwan et al. reported that only AITC-sensitive neurons that also respond to TRPV1 express TRPA1 [42]. Even though we did not further address the specific target of 8,9-EET in this TRPA1-expressing cell population, multiple studies have shown that calcium transients in nociceptive neurons induce plasticity changes through the activation of PKCε, p38 MAPK or ERK which result in reduced activation thresholds and increased firing rates [43]. It could be speculated that 8,9-EET may directly activate TRPA1 at high concentrations although various other possible targets exist. However, due to its electrophilic character, 8,9-EET can potentially sensitize TRPA1 by direct interaction with its intracellular cysteins as previously described for other lipids like 4-HNE or cyclopentons [44,45]. In addition to a potential direct activation of the TRPA1-positive cell population, we found that lower doses of 8.9-EET potentiate AITC-induced calcium flux. Here, 8,9-EET may modulate TRPA1 indirectly via G-protein coupled receptors as described for bradykinin [46]. Moreover, to increase functionality of certain TRP-channels such as TRPC6, EETs have already been shown to promote membrane translocation [13]. Finally, other TRPA1 independent downstream processes, such as sensitized voltage gated calcium channels or calcium transporters may be involved in the observed increased calcium responses.

We also investigated whether 8,9-EET modulates the activation of TRPA1-expressing neurons. 8,9-EET application to isolated sciatic nerves caused an increased neuropeptide release in response to AITC. Peripheral nerve axons resemble peripheral sensory terminals in their common properties of sensory and signal transduction and CGRP neuropeptides are stored all along axons of small diameter peptidergic neurons [47]. Stimulation of those cells by AITC induces a translocation of TRPA1 to the membrane where it can be activated resulting in calcium influx, subsequent vesicle fusion and neurotransmitter release. CGRP release from sciatic nerves can be sensitized by activation of G-protein-coupled receptors and related protein kinases [47] and that CGRP can induce mechanical hyperalgesia and central sensitization [48]. TRPA1 expressing neurons appear to mediate mechanotransduction and blockade of TRPA1 attenuates the development of mechanical hyperalgesia [27,42,49]. Our finding that 8,9-EET increases CGRP release from TRPA1 expressing neurons strongly suggests that sEH activity in primary afferents may prevents mechanical hypersensitivity.

In keeping with this, 8,9-EET injection into a hind paw lowered the mechanical but not thermal threshold in wild- type mice. Wild- type mice recover from zymosan-induced mechanical hyperalgesia 2 to 4 days after injection, a time during which 8,9-EET levels decrease in the paw tissue. Our finding that 8,9-EET sensitizes primary afferents and reduces mechanical thresholds suggested that hydrolysis of 8,9-EET could potentially contribute to the resolution of mechanical hyperalgesia during inflammation. In accordance with this hypothesis, sEH-deficient mice, which exhibit elevated 8,9-EET levels during inflammation, show a dramatically reduced recovery from mechanical hyperalgesia.

## Conclusion

sEH is expressed in sensory ganglia where it contributes to the metabolism of 8,9-EET which can sensitize nociceptors. As a consequence of genetic deletion of sEH mice exhibit exaggerated hyperalgesia during inflammation underlining the importance of the antinociceptive function sEH. Pharmacological interventions influencing the EET-pathway, which are actually highly considered to treat cardiovascular diseases, should therefore be taken with care in terms of pain side effects.

## Methods

### Animals

sEH^-/- ^mice [50] were crossbred for 10 generations onto the C57BL/6 background. In all experiments the ethical guidelines for investigations in conscious animals of the NIH and International Association for the Study of Pain were followed, and the procedures were approved by the local Ethics Committee. sEH^-/- ^mice were compared with strain-, age-, and sex-matched C57BL/6 control mice.

### Behavioral tests

To determine mechanical hyperalgesia mice were kept in test cages for 2 h on day one for habituation. On day two, baseline paw withdrawal latencies (PWL) in response to mechanical stimulation were determined. Briefly, animals were placed on an elevated wire grid and the plantar surface of the paw stimulated using a Dynamic Plantar Aesthesiometer (Ugo Basile, Comerio VA, Italy). We used a force increasing by 0.5 g every second with an upper limit of 5 g. Paw withdrawal latencies were measured in sec ± 0.1 with a cut off of 20 sec.

To test the effects of EETs on mechanical hyperalgesia, 20 μl of either 8,9-, 11,12- or 14,15-EET (10 μM) were injected subcutaneously into the mid plantar side of the left hind paw. Control animals received a corresponding volume of ethanol (3,2% v/v). Mechanical hyperalgesia was assessed from 0.5 to 4 h after EET- injection using the Dynamic Plantar Aesthesiometer.

Mechanical hyperalgesia after zymosan-induced inflammation has been described previously [19,23]. 20 μl of a zymosan A (Sigma, Deisenhofen, Germany) suspension (12.5 mg/ml in phosphate buffered saline) was injected subcutaneously into the plantar side of one hind paw. Mechanical hyperalgesia was assessed from 0.5 h to 96 h after zymosan injection using the Dynamic Plantar Aesthesiometer as described above.

To exclude gender differences only male mice were used. The non-injected and injected paws were measured alternately at intervals of 5-10 min. For all behavioral tests the observer was unaware either of genotypes or treatment.

### Western blot analysis

For Western blot analysis we dissected skin tissue from the mid-plantar region of the paw (1-3 mm deep) and collected DRGs from L4-6 segments. Tissues were homogenised and sonicated in PBS and whole cell lysates containing 30 μg protein used for separation on a 15% SDS-polyacrylamide gel. After blotting, COX-2 was detected with a polyclonal antibody (1:500) from Cayman (Ann Arbor, MI). sEH was detected with a polyclonal anti-mouse sEH antibody (dilution of 1:2000) raised against recombinant murine sEH produced in a baculovirus expression system, and then purified to apparent homogeneity by affinity chromatography. Antibodies against ß-actin (1:5000), HSP90 (1:2000) or ERK-2 (1:500) (all from Santa Cruz, CA) were used to control for loading.

### Immunohistochemistry using Multi-epitope ligand cartography (MELC)

DRGs and paws (48h after zymosan) were dissected from mice intracardially perfused with 0.9% saline followed by 4% PFA/PBS (pH 7.4). After 24 h incubation in 30% sucrose/PBS the tissue was cryostat sectioned at 10 μm and stored at 4° C until use.

To analyse sEH expression we combined conventional immunohistochemistry for sEH staining with the MELC-technique using FITC-labelled antibodies against cell type specific markers [19,20]. For sEH-staining we blocked the slices with 10% BSA and 1% mouse serum in PBS followed by a 15 h incubation with a polyclonal rabbit anti sEH antibody (1:1000) diluted in the blocking solution and a 1 h incubation with a anti-rabbit-Cy3 antibody (1:1000) (Sigma, Deisenhofen, Germany) diluted in 1% BSA/PBS. To compare the sEH signal between wt and sEH^-/- ^mice the DRG slices were costained with NeuN and DAPI [23]. For comparison with multiple markers the slices stained for sEH were then transferred to the MELC-system. Here, we used monoclonal anti-neuronal nuclei (NeuN) (Millipore, Billerica, MA), monoclonal anti NF200 (AbD Serotec, Oxford, UK), monoclonal anti glial fibrillary acidic protein (GFAP) (Sigma, Deisenhofen, Germany) and Isolectin B4 (IB4) (Sigma, Deisenhofen, Germany) which were all directly labelled with FITC as described [19,51].

### Primary dorsal root ganglia (DRG)-cultures

DRGs were dissected from all spinal segments of adult mice and transferred to ice cold HBSS with CaCl_2 _and MgCl_2 _(Invitrogen, Carsbad, CA, USA). For dissociation, isolated DRGs were treated for 90 min with collagenase/dispase (500 U/ml collagenase; 2.5 U/ml dispase) followed by a 20 minutes incubation with 0.05% Trypsin (Invitrogen, Carsbad, CA, USA). After removal of the enzyme-solutions, cells were washed twice with neurobasal medium containing 10% FCS respectively. Then cells were mechanically dissociated by pipetting (Gilson 1000 μl) and plated on culture dishes or poly-l-lysine (Sigma, Deisenhofen, Germany) coated glass cover slips for calcium imaging. After two hours incubation, neurobasal medium containing 2 mM L-glutamine, 100 U/ml penicillin, 100 μg/ml streptomycin, 50 μg/ml gentamicin and supplement B27 (all from Invitrogen, Carsbad, CA, USA) were added and cells incubated for 24-48 h at 37° C without serum, NGF or any other neurotrophins.

### Calcium imaging experiments

Calcium imaging experiments were performed on DRG cultures 24 h to 48 h after preparation. Cells were loaded with 5 μM Fura-2-AM-Ester containing 0.02% Pluronic F-127 (both from Biotrend, Köln, Germany) and incubated for 30 to 45 min at 37° C. For baseline measurements extracellular solution (145 mM NaCl,1.25 mM CaCl_2_, 1 mM MgCl_2_, 5 mM KCl, 10 mM D-glucose, 10 mM HEPES, pH 7.3) was added by bath application at a flow-rate of 1-2 ml/min. EETs were dissolved in the extracellular solution at a concentration of 1 and 10 μM. Cells were stimulated for 30 seconds. For control the corresponding volume of ethanol was applied in the same way. At the end of each measurement, cells were stimulated with 40 mM KCl for 30 seconds to identify viable neurons. Images were taken with an Axioscope 2 upright microscope (Zeiss, Jena, Germany) using a 10x Achroplan water immersion objective (Zeiss). The microscope was equipped with an Imago CCD camera, a Polychrome IV monochromator (all TILL Photonics, Gräfelfing, Germany). Images were acquired and processed using Tillvision software [52]. Quantitative characterization of 8,9-EET responding cells was done using a Nikon Ti-E PFS Large Research Microscope cells were recorded every 8 sec. with a QImaging EXI Aqua Digital Camera (Q-imaging, Surrey, BC, Canada) after alternating excitation with 340nm and 380nm by a Ti-FL Epi-Fluorescence Illuminator (Sutter Instrument Company Novato, CA) regulated by a SmartShutter Controller Unit (Sutter Instrument Company Novato, CA). Emission was filtered by an ET FURA-2 Hybrid Filter. Extracellular solutions were applied via a 360 μm perfusion pencil tip and a valve bank control system (both from AutoMate scientific inc., Berkeley, CA) with » 1 drop/sec gravity flow. Fluorescence intensities of single cells were calculated by the NIS-Elements (AR Advance Research Acquisition and analysis) software and transferred to Microsoft Excel for further statistical analysis.

The DRG neurons were stimulated for 10 sec with 10 μM 8,9-EET, 200 μM menthol, 0.3 μM capsaicin, 100 μM AITC and 40 mM KCL. Neurons from 2 different dissections and 6 different experiments were used for calculations. For sensitization experiments, DRG cells were stimulated with 50 μM AITC for 15 seconds twice with an interval of 10 minutes. 1 μM 8,9-EET were perfused over the cells for two minutes prior to the second AITC-stimulation.

### Sciatic nerve CGRP-release measurements

The experimental procedures were performed exactly as described before [47]. C57BL/6 mice were sacrificed in CO_2_-. Sciatic nerves were excised and continuously rinsed with synthetic interstitial fluid (SIF, Bretag, 1969) consisting (in mM) of 107.8 NaCl, 26.2 NaCO_3_, 9.64 Na-gluconate, 7.6 sucrose, 5.05 glucose, 3.48 KCl, 1.67 NaH_2_PO_4_, 1.53 CaCl_2 _and 0.69 MgSO_4_, gassed with 95% oxygen and 5% carbon dioxide creating pH 7.4. Preparations were placed in SIF inside a thermostatic shaking bath at 32°C for a 30 min washout-phase before the experiment. All experiments were performed with matched pairs of a treated and untreated side from the same animal. An experiment consisted of four incubations of 5 minutes in mesh wells filled with 100 μl carbogen-saturated SIF. After two incubations to determine basal CGRP release and spontaneous variation at 32°C, the samples were stimulated with AITC (50 μM). 10 μM 8,9-EET was added in steps two to four. Immediately after removal of the nerve from an incubation tube a sample volume of 100 μl was processed using a CGRP-EIA kit (SPIbio, France) [53]. The CGRP detection level of the method is ~2 pg/ml.

### Determination of *EETs *and their metabolites by liquid chromatography-tandem mass spectrometry (LC/MS-MS)

#### Sampling of tissue from DRGs and inflamed paws

For the determination of EETs and prostanoids during inflammation, 20 μl of 10 mg/ml Zymosan A was injected intraplantarily into hind paws of wild type and sEH-deficient mice. At indicated time points, DRGs from L4-6 segments and the tissue from the mid-plantar region of the paw (1- 3 mm deepma) was dissected, weighed and directly transferred to organic extraction solvents including 20 μl internal standards (5,6 EET-d11, 8,9 EET-d8, 11,12 EET-d8 and 14,15 EET-d8 all with a concentration of 200 ng/ml in methanol) and stored at -80° C. The next day, tissue samples were homogenized in extraction solvents using a Retsch Mixer-Mill MM 200 (Retsch, Haan, Germany).

#### Sampling of conditioned media from DRG cultures

For the determination of EET-release from sensory neurons we used primary" DRG-neurons from wild type and sEH-deficient mice. After 24 h in culture, cells were stimulated with 0.5 μM arachidonic acid and incubated for 2 h. Then, the cell culture supernatant was removed, internal standards added, and samples directly extracted.

#### Sample extraction and standards

All standard substances were obtained from Cayman Chemical, Ann Arbor, MI, USA. Stock solutions with 2500 ng/ml of all analytes were prepared in methanol. Working standards were obtained by further dilution with a concentration range of 0.1-250 ng/ml for *EETs *respectively.

Sample extraction was performed with liquid-liquid-extraction. Therefore tissue or cell culture medium was extracted twice with ethyl acetate. The combined organic phases were removed at a temperature of 45° C under a gentle stream of nitrogen. The residues were reconstituted with 50 μl of methanol/water/(50:50, v/v), centrifuged for 2 minutes at 10,000x g and then transferred to glass vials (Macherey-Nagel, Düren, Germany) prior to injection into the LC-MS/MS system. Extraction and LC-MS/MS-instrumentation for measurment of prostanoids was described previously [19,23].

#### Instrumentation for measuring 5,6-EET, 8,9-EET, 11,12-EET, 14,15-EET and their dehydro metabolites

The LC/MS-MS system comprised an API 4000 triple quadrupole mass spectrometer (Applied Biosystems, Darmstadt, Germany), equipped with a Turbo-V-source operating in negative ESI mode, an Agilent 1100 binary HPLC pump and degasser (Agilent, Waldbronn, Germany) and an HTC Pal autosampler (Chromtech, Idstein, Germany) fitted with a 25 μL LEAP syringe (Axel Semrau GmbH, Sprockhövel, Germany). High purity nitrogen for the mass spectrometer was produced by a NGM 22-LC/MS nitrogen generator (cmc Instruments, Eschborn, Germany).

For the chromatographic separation a Gemini NX C18 column and precolumn were used (150 mm × 2 mm i. d., 5 μm particle size and 110Å pore size from Phenomenex, Aschaffenburg, Germany). A linear gradient was employed at a flow rate of 0.5 ml/min mobile phase with a total run time of 17.5 minutes. Mobile phase A was water/ammonia (100:0.05, v/v) and B acetonitrile/ammonia (100:0.05, v/v).

The gradient started from 85% A to 10% within 12 min. This was held for 1 min at 10% A. Within 0.5 min the mobile phase shifted back to 85% A and was held for 3.5 min to equilibrate the column for the next sample. The injection volume of samples was 20 μl.

Quantification was performed with Analyst Software V 1.4.2 (Applied Biosystems, Darmstadt, Germany) employing the internal standard method (isotope- dilution mass spectrometry). Ratios of analyte peak area and internal standard area (y-axis) were plotted against concentration (x-axis) and calibration curves were calculated by least square regression with 1/concentration^2 ^weighting.

## Competing interests

The authors declare that they have no competing interests.

## Authors' contributions

CB, KS, GG and CWJ conceived and designed the experiments. MS performed the behavior, calcium imaging, histology, tissue sampling and CGRP release experiments. CB performed Western blot, histology and calcium imaging experiments. OC performed behavior experiments. KA performed Western blot experiments. IF and RPB provided the sEH knockout mice and sEH antibodies. CA and HS performed the LC-MS/MS measurements. PWR and MJF introduced the CGRP-release experiments. CB, KS, MS and CJW wrote the manuscript. All authors read and approved the final manuscript.
